# The Absolute Risk of Venous Thrombosis after Air Travel: A Cohort Study of 8,755 Employees of International Organisations

**DOI:** 10.1371/journal.pmed.0040290

**Published:** 2007-09-25

**Authors:** Saskia Kuipers, Suzanne C Cannegieter, Saskia Middeldorp, Luc Robyn, Harry R Büller, Frits R Rosendaal

**Affiliations:** 1 Department of Clinical Epidemiology, Leiden University Medical Center, Leiden, The Netherlands; 2 Department of Vascular Medicine, Academic Medical Center, Amsterdam, The Netherlands; 3 Nestlé Medical Services, Vevey, Switzerland; 4 Einthoven Laboratory for Experimental Vascular Medicine, Leiden University Medical Center, Leiden, The Netherlands; McGill University, Canada

## Abstract

**Background:**

The risk of venous thrombosis is approximately 2- to 4-fold increased after air travel, but the absolute risk is unknown. The objective of this study was to assess the absolute risk of venous thrombosis after air travel.

**Methods and Findings:**

We conducted a cohort study among employees of large international companies and organisations, who were followed between 1 January 2000 and 31 December 2005. The occurrence of symptomatic venous thrombosis was linked to exposure to air travel, as assessed by travel records provided by the companies and organisations. A long-haul flight was defined as a flight of at least 4 h and participants were considered exposed for a postflight period of 8 wk. A total of 8,755 employees were followed during a total follow-up time of 38,910 person-years (PY). The total time employees were exposed to a long-haul flight was 6,872 PY. In the follow-up period, 53 thromboses occurred, 22 of which within 8 wk of a long-haul flight, yielding an incidence rate of 3.2/1,000 PY, as compared to 1.0/1,000 PY in individuals not exposed to air travel (incidence rate ratio 3.2, 95% confidence interval 1.8–5.6). This rate was equivalent to a risk of one event per 4,656 long-haul flights. The risk increased with exposure to more flights within a short time frame and with increasing duration of flights. The incidence was highest in the first 2 wk after travel and gradually decreased to baseline after 8 wk. The risk was particularly high in employees under age 30 y, women who used oral contraceptives, and individuals who were particularly short, tall, or overweight.

**Conclusions:**

The risk of symptomatic venous thrombosis after air travel is moderately increased on average, and rises with increasing exposure and in high-risk groups.

## Abstract 2

In a cohort study of 8,755 employees of large international organizations followed for 38,910 person-years, Suzanne Cannegieter and colleagues find a risk of one thrombosis per 4,656 long-haul flights.

## Editors' Summary

**Background.:**

Blood normally flows smoothly throughout the human body, supplying the brain and other vital organs with oxygen and nutrients. When an injury occurs, proteins called clotting factors make the blood gel or coagulate at the injury site. The resultant blood clot (thrombus) plugs the wound and prevents blood loss. Sometimes, however, a thrombus forms inside an uninjured blood vessel and partly or completely blocks the blood flow. A clot inside one of the veins (vessels that take blood to the heart) deep within the body is called a deep vein thrombosis (DVT). Symptoms of DVT (which usually occurs in the deep veins of the leg) include pain, swelling, and redness in one leg. DVT is usually treated with heparin and warfarin, two anticoagulant drugs that stop the blood clot growing. If left untreated, part of the clot (an embolus) can break off and travel to the lungs, where it can cause a life-threatening condition called pulmonary embolism (PE). Fortunately, DVT and PE are rare but having an inherited blood clotting disorder, taking an oral contraceptive, and some types of surgery are all risk factors for them. In addition, long-haul plane travel increases the risk of DVT and PE, known collectively as venous thrombosis (VT) 2- to 4-fold, in part because the enforced immobilization during flights slows down blood flow.

**Why Was This Study Done?:**

Although the link between air travel and VT was first noticed in the 1950s, exactly how many people will develop DVT and PE (the absolute risk of developing VT) after a long flight remains unknown. This information is needed so that travelers can be given advice about their actual risk and can make informed decisions about trying to reduce that risk by, for example, taking small doses of anticoagulant medicine before a flight. In this study, the researchers have determined the absolute risk of VT during and after long-haul air travel in a large group of business travelers.

**What Did the Researchers Do and Find?:**

The researchers enrolled almost 9,000 employees from several international companies and organizations and followed them for an average of 4.4 years. The details of flights taken by each employee were obtained from company records, and employees completed a Web-based questionnaire about whether they had developed VT and what risk factors they had for the condition. Out of 53 thrombi that occurred during the study, 22 occurred within eight weeks of a long-haul flight (a flight of more than four hours). From this and data on the total time employees spent on long-haul flights, the researchers calculated that these flights tripled the risk of developing VT, and that the absolute risk (the probability of something occurring in a certain time period) of a VT occurring shortly after such travel was one event per 4,656 flights. They also calculated that the risk of VT was increased by exposure to more flights during a short period and to longer flights and was greatest in the first two weeks after a flight. In addition, the risk of VT was particularly high in young employees, women taking oral contraceptives, and people who were short, tall or overweight.

**What Do These Findings Mean?:**

The main finding of this study is that the absolute risk of VT after of a long-haul flight is low—only one passenger out of nearly 5,000 is likely to develop VT because of flying. However, the study included only healthy people without previous VT whose average age was 40 years, so the absolute risk of VT after long-haul flights might be higher in the general traveling population. Even so, this finding strongly suggests that prophylactic (preventative) use of anticoagulants by all long-haul travelers may not be justified because these drugs have potentially dangerous side effects (for example, they can cause uncontrolled bleeding). Subgroups of travelers with additional risk factors for VT might, however, benefit from the use of this and other prophylactic measures, but randomized trials are needed to find out who would benefit most from which prophylactic measure.

**Additional Information.:**

Please access these Web sites via the online version of this summary at http://dx.doi.org/10.1371/journal.pmed.0040290.

- MedlinePlus encyclopedia pages on blood clots, deep vein thrombosis, and pulmonary embolism (in English and Spanish)
- Information from the US National Heart Lung and Blood Institute on deep vein thrombosis, including an animation of how DVT causes pulmonary embolisms
- Information for patients from the UK National Health Service Direct health encyclopedia on deep vein thrombosis (in several languages)
- Information for travelers on DVT from the US Centers for Disease Control and Prevention and from the UK National Travel Health Network and Centre
- This study came out of the WHO Research Into Global Hazards of Travel (WRIGHT) project, and WHO's WRIGHT project on Air Travel and Venous Thromboembolism, of which his study forms a part, has a Web site

## Introduction

In 1951, Jacques Louvel reported four cases of venous thrombosis following air travel [1]. More recently, several investigators have shown an association between air travel and venous thrombosis, with a 2- to 4-fold increased risk in most studies [2–8]. Two follow-up studies demonstrated a dose–response relationship between the occurrence of pulmonary embolism shortly after arrival at the airport and the distance travelled [9,10]. Still, the most relevant element, i.e., the absolute risk of symptomatic venous thrombosis after long-distance air travel, remains unknown. One follow-up study demonstrated an absolute risk of severe pulmonary embolism occurring shortly after arrival of 1/200,000 passengers [9], whereas another study showed a risk of fatal pulmonary embolism of 1.3 per million passengers [11]. Asymptomatic clots have been found in 1% to 10% of air travellers [12–14]. Hence, the absolute risk of symptomatic venous thrombosis after long-haul travel must lie between these extremes.

Knowledge of the absolute risk of symptomatic thrombosis after air travel is needed to provide travellers with solid advice regarding their actual risk and to evaluate the utility of prophylactic measures. Since two billion passengers fly annually (2005 data [15]), even a small increase in risk will have a major impact on the number of events. Overestimation of the risk may lead to inappropriate use of potentially dangerous antithrombotic drugs [16,17].

In addition to estimating the absolute risk of symptomatic deep vein thrombosis or pulmonary embolism after long haul air travel, we assessed the effects of exposure to several flights within a short time frame, duration of travel, and the occurrence of venous thrombosis in relation to the time passed after air travel. Finally, we determined the effect of air travel within high-risk groups.

## Methods

### Study Design

We performed a cohort study among employees of large international companies and organisations. During the follow-up period, thrombotic events were linked to exposure to air travel.

### Participating Companies and Organisations

Participating companies and organisations were Nestlé (Vevey, Switzerland), General Mills (Minneapolis, Minnesota, US), the US Centers for Disease Control and Prevention (Atlanta, Georgia, US), the World Bank and the International Monetary Fund (Washington, D. C., US), Shell Companies based in The Hague (The Netherlands) and London (UK), Shell Exploration and Production (SIEP) based in Rijswijk (The Netherlands), Sakhalin Energy Investment Company Ltd (SEIC) based in Sakhalin (Russia), and TNT NV (Thomas Nationwide Transport, Hoofddorp, The Netherlands). All organisations and companies had a central database with records of employees' business travel. Start of follow-up varied per company, between 1 January 1998 and 1 January 2001 or at start of the employment if later. Follow-up ended between 1 December 2002 and 1 January 2006, when venous thrombosis was diagnosed or at the end of employment, whichever occurred first, with approximately 5 y of follow-up per company.

### Questionnaires and Flight Data

We developed Web-based questionnaires, using Apian Survey Pro 3.0. These contained questions about venous thrombosis occurrence (at any time point in the follow-up period) and risk factors for venous thrombosis. Employees were invited to take part by a personal e-mail containing a link to the questionnaire and a unique password, which ensured that each individual could enter only once. With intervals of a few weeks, nonresponding employees received two or three reminders. The questionnaire can be viewed at http://www.clinicalresearch.nl/epidemiology/wrightquestionnaire/wrightquestionnaire.asp (note: a password is not necessary to view the questionnaire).

Date of travel and duration of travel (not including stopover time) were taken from the organisations' travel database.

### Outcomes

Participants who reported venous thrombosis were asked to fill in a consent form for medical chart review. Only symptomatic first venous thrombotic events that were diagnosed with objective methods were considered. Deep vein thrombosis had to be diagnosed by compression ultrasonography or venography. Pulmonary embolism had to be diagnosed by spiral CT scanning, high-probability ventilation-perfusion scanning, or angiography. Superficial thrombophlebitis was not included.

### Statistical Analysis

For the analysis of the overall effect of flying, exposure time was defined as a time window of 8 wk after a long-haul flight (flight of at least 4 h). For each individual, the total time exposed and not exposed was calculated. The incidence rate (IR) of venous thrombosis within 8 wk of a long-haul flight was calculated by dividing the number of cases that occurred in this exposure window by the number of exposed person-years (PY). The IR of venous thrombosis without exposure was calculated in the same way (events over person-time outside exposure windows). The incidence rate ratio (IRR) adjusted for age and sex was calculated using Poisson regression analysis. The overall effect of flying was assessed for the whole group of employees and separately for subgroups based on sex, age, oral contraceptive use, body mass index (BMI), and height. The number of person-years exposed and unexposed to oral contraceptive use was calculated for women younger than 50 y.

In addition, we calculated the absolute risk of venous thrombosis per flight by dividing the number of cases that occurred within 8 wk of a long-haul flight by the total number of flights longer than 4 h made by all responding employees.

**Figure 1:**
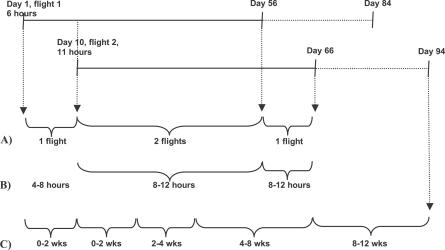
Example of the Calculation of Person-Years of Exposure in Three Different Ways An employee makes one flight of 6 h on day 1 and another flight of 11 h on day 10. (A) Per number of flights: from day 1 to 10, this employee is exposed to only one flight. Exposure from day 10 to 56, is two flights; from day 56 to 66, one flight. (B) Per category of duration: from day 1 to 10 the employee is exposed to one flight of 6 h (so 10 d in the category of 4–8 h); from day 10 to 56, to two flights, of which the longest is 11 h (so 46 d in the category of 8−12 h); from day 56 to 66, one flight of 11 h (so again 10 d in the category of 8–12 h). (C) Per time window. From day 1 to 10, the employee is exposed to the time window of 0–2 wk due to the first flight. At day 10, the time is “reset,” so from day 10 to 24, the employee is exposed to the time window of 0–2 wk again. From day 24 to 38, the employee is exposed to the time window of 2–4 wk, from day 38 to 66 the time window of 4–8 wk and, finally, from day 66 to 94 the time window of 8–12 wk.

Employees were often exposed to more than one flight in the 8 wk exposure windows, so time windows were frequently overlapping. To assess the effect of number of flights, the total time employees were exposed to one to five flights or more was calculated. Thus, IRs and IRRs for exposure to one or two, three or four, and five or more flights could be calculated (Figure 1A). Furthermore, we calculated the increase in risk for each extra flight using Poisson regression.

To assess the effect of duration of travel, we calculated IRs and IRRs within 8 wk of flights of varying duration, i.e., 0–4 h, 4–8 h, 8–12 h, 12–16 h, and longer than 16 h. If time windows were overlapping, only the duration of the longest flight was considered for this analysis (Figure 1B). The absolute risk per flight for each category of duration was calculated by dividing the number of cases that occurred within 8 wk of a flight by the total number of flights in the corresponding category. Furthermore, we calculated the increase in risk for each extra hour of duration of a flight using Poisson regression.

The occurrence of venous thrombosis in relation to the period of time that had passed after travelling was assessed by calculating IRs and IRRs for periods of 0–2 wk, 2–4 wk, 4–8 wk, and 8–12 wk after a flight of at least 4 h. The period of 12 wk after a flight was split into these four time windows, creating mutually exclusive exposure windows. If a person was exposed to several flights and hence to more than one time window, the overlapping time was counted only in the time window closest to the flight (Figure 1C).

## Results

A total of 27,496 employees were invited to participate and 8,755 questionnaires were completed, yielding an overall response of 32% (range per organisation 15%–80%). General characteristics of the study population are shown in Table 1. More than half of the responders (*n* = 4,915, 56%) were men and the mean age was 40 y. The total follow-up time of participating employees was 38,910 PY, with a mean follow-up per participant of 4.4 y.

**Table 1:**
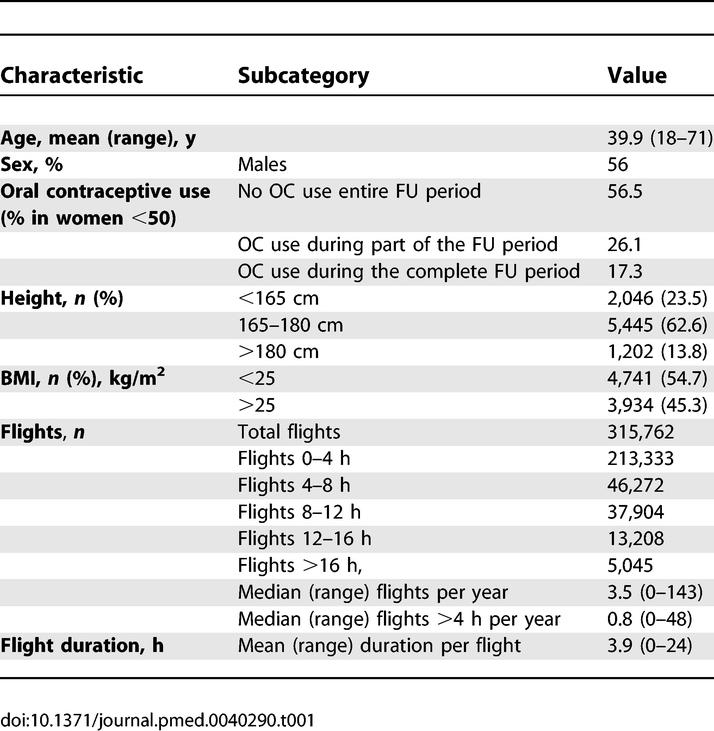
General Characteristics of the Study Population and Flight Data

### Flight Data

Flight data are shown in Table 1. The 8,755 responders had made 315,762 flights during follow-up, and 6,440 individuals had travelled by air at least once. Approximately one-third of all flights were long-haul flights (at least 4 h, *n* = 100,208). The mean number of long-haul flights per person per year was 2.6 (range 0–48, median 3.5).

### Thrombotic Events

In total, 76 employees reported that they had suffered from venous thrombosis in the follow-up period. Of these 76 possible cases 23 were not validated: four employees did not give permission for medical chart review, two doctors could not be traced, six appeared to have suffered from an arterial event (such as myocardial infarction), and 11 had been diagnosed with superficial thrombophlebitis. The remaining 53 employees all had an objectively confirmed venous thrombotic event. Deep vein thrombosis of the leg was diagnosed in 34, pulmonary embolism in nine, a combination in eight, and deep vein thrombosis of the arm in two.

### Absolute Risks and Incidence Rate Ratios

The overall IR of venous thrombosis was 1.4/1,000 PY (95% confidence interval [CI] 1.0–1.8/1,000 PY). The total time employees were exposed to a postflight period of 8 wk added up to 6,872 PY when only flights longer than 4 h were considered. Within 8 wk of a long-haul flight 22 events occurred, yielding an IR of 3.2/1,000 PY (95% CI 2.0–4.7/1,000 PY). The time the employees were not exposed to any flight (long- or short-haul) was 27,772 PY, during which 29 cases occurred, yielding an IR of 1.0/1,000 PY (95% CI 0.7–1.5/1,000 PY). Thus, the IRR was 3.2 (95% CI 1.8–5.6). The total number of long-haul flights made by the employees was 102,429, hence the absolute risk of venous thrombosis was 21.5/100,000 flights, or 1/4,656 flights.

Both the unexposed incidence rate and the incidence rate in the exposed were higher in women (1.3/1,000 PY and 4.4/1,000 PY) than in men (0.8/1,000 PY and 2.7/1,000 PY), so their IRRs were approximately the same (Table 2). Although the IR of venous thrombosis in the unexposed group increased with age, in the individuals exposed to air travel it was highest in the youngest age category (4.9/1,000 PY, 95% CI 0.9–12.1/1,000 PY) and lowest in the oldest (2.9/1,000 PY, 95% CI 0.7–6.5/1,000 PY), and hence the IRR decreased with age, with an IRR of 7.7 (95% CI 1.6–38.4) for those under 30 (Table 2).

**Table 2:**
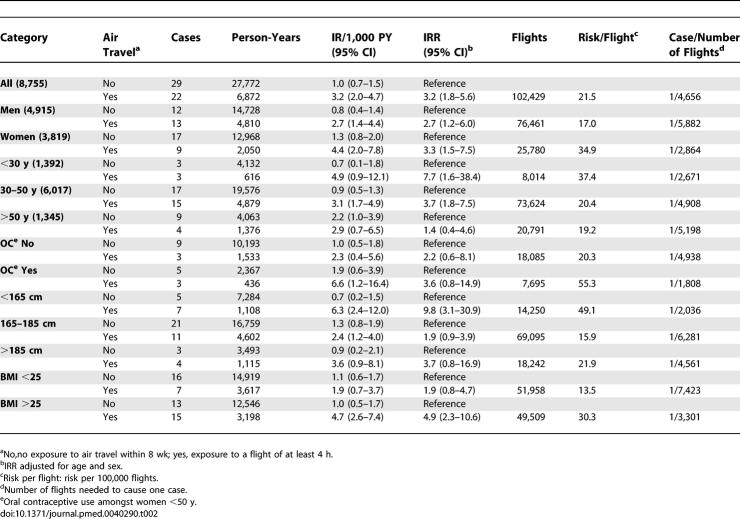
Incidence Rates, Absolute Risks, and Incidence Rate Ratios within 8 Weeks of Long-Haul Flights, for the Whole Study Population and Stratified by Sex, Age, Oral Contraceptive Use, Height, and BMI

Women using oral contraceptives had an increased risk of venous thrombosis, both at baseline (IR 1.9, 95% CI 0.6–3.9) and after long distance flights (IR 6.6, 95% CI 1.2–16.4). Thus, the IRR of exposure to air travel was higher in women using oral contraceptives (3.6, 95% CI 0.8–14.9) than in women not using oral contraceptives (2.2, 95% CI 0.6–8.1).

The baseline IR of venous thrombosis did not differ much between subgroups based on height. The IR after air travel was highest in individuals shorter than 165 cm (IR 6.3/1,000 PY, 95% CI 2.4–12.0/1,000 PY) and lowest in those between 165 and 185 cm (IR 2.4/1,000 PY, 95% CI 1.2–4.0/1,000 PY). In employees taller than 185 cm, the IR after air travel was 3.6/1,000 PY (95% CI 0.9–8.1/1,000 PY). Hence, the IRR was highest in the shortest employees (IRR 9.8, 95% CI 3.1–30.9).

BMI did not affect the baseline thrombosis risk, but the IR after air travel was higher in employees with a BMI over 25 kg/m^2^ (4.7/1,000 PY, 95% CI 2.6–7.4/1,000 PY) than in those with a BMI lower than 25 kg/m^2^ (1.9/1,000 PY, 95% CI 0.7–3.7/1,000 PY).

The risk of venous thrombosis increased with the number of flights per employee, as shown in Table 3. When someone was exposed to only one or two long-haul flights, the IR per 1,000 PY was 2.6 (95% CI 1.4–3.2), which tripled after exposure to five or more long-haul flights. With each extra flight the employees were exposed to, the risk increased 1.4-fold (95% CI 1.2–1.6).

**Table 3:**
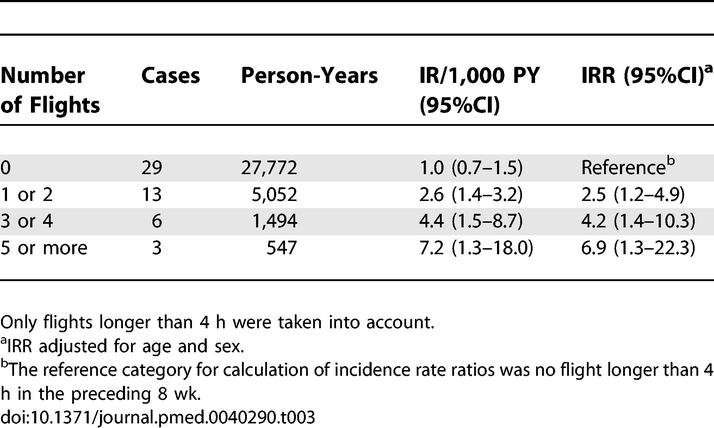
Incidence Rates and Incidence Rate Ratios for Exposure to an Increasing Number of Flights

The effect of duration of travel is shown in Table 4. The IR increased from 0.5/1,000 PY (95% CI 0–1.4/1,000 PY) when employees had travelled less than 4 h to 5.9/1,000 PY (95% CI 1.5–13.4/1,000 PY) when they had travelled for more than 16 h. For each extra hour of flight duration, the IRR increased 1.1-fold (95% CI 1.1–1.2). Expressed as risk per number of flights, the risk increased from 1/106,667 flights for flights shorter than 4 h up to 1/1,264 for flights longer than 16 h.

**Table 4:**
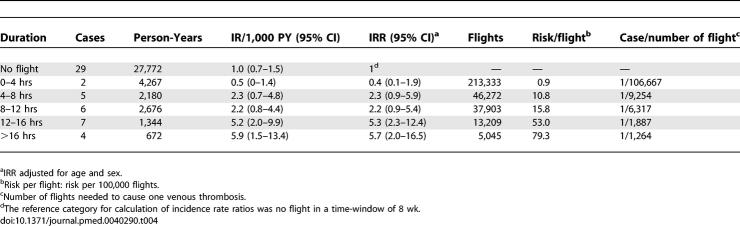
Incidence Rates and Incidence Rate Ratios after Flights of Increasing Duration

In Table 5, the IRs and IRRs are shown in relation to the time that had elapsed after travelling. In the first 2 wk after a long-haul flight, the risk of venous thrombosis was highest, with an IR per 1,000 PY of 4.7 (95% CI 2.4–7.7). The risk gradually decreased with time and returned to the baseline risk after 8 wk.

**Table 5:**
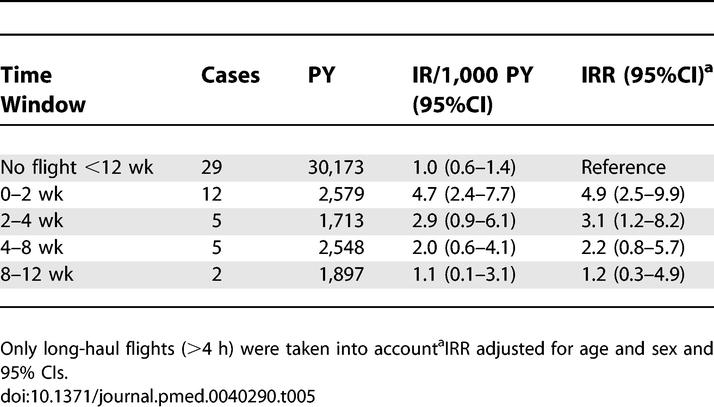
Incidence Rates and Incidence Rate Ratios in Varying Time Windows after Long-Haul Flights

## Discussion

In this follow-up study, we found an overall absolute risk of symptomatic venous thrombosis of 1/4,656 passengers within 8 wk after flights longer than 4 h. This risk is equivalent to an IR of 3.2/1,000 PY. The risk was 3.2-fold increased compared to those who did not travel by air. The risk of venous thrombosis increased with exposure to several flights and longer duration of travel, and it decreased with time after a flight. It was particularly high in younger travellers, women (especially those taking oral contraceptives), individuals who were particularly short (<165 cm) or tall (>185 cm), and those with a BMI over 25 kg/m^2^, although due to the small number of cases, some confidence intervals were wide, indicating considerable uncertainty for the effect estimates.

The observed rate ratio of 3.2 for flights longer than 4 h is similar to the odds ratios found in most case-control studies [3,4,7,8]. Only two studies have previously described absolute risks of venous thrombosis after air travel, but only for severe pulmonary embolism occurring immediately after flying [9,10]. In our study, the risk of venous thrombosis was highest in the first 2 wk after air travel, which was also demonstrated by Kelman in a record-linkage study [5].

Previous studies showed a dose–response relationship between the distance travelled and the risk of venous thrombosis [9,10]. In our study we observed three dose–response relationships. The risk of venous thrombosis increased with (1) duration of air travel and (2) number of flights, and (3) decreased with time after the flight. These dose–response relationships are in line with a causal association between air travel and deep vein thrombosis.

The effect of air travel was pronounced in women using oral contraceptives. This synergy of oral contraceptive use and air travel was also demonstrated in a previous case-control study [7]. Travellers who were shorter than 165 cm or taller than 185 cm also had a higher risk of venous thrombosis after air travel than those with a height between 165 and 185 cm. In the tall travellers, this may be explained by an extremely cramped position due to insufficient leg-space. In travellers who are shorter than 165 cm, the increased risk may be explained by pressure on the poplitial vein by the airplane seat, when their feet do not touch the floor. This higher risk in both tall and short travellers was previously found in a large population-based case-control study [3]. This is an important finding that has now been demonstrated in two different populations, indicating a need for adjustable seating in aircraft.

A remarkable finding in our study was that the IR of venous thrombosis after exposure to flights shorter than 4 h seemed lower (0.5/1,000 PY) than the unexposed IR (1.0/1,000 PY). Although the confidence intervals were wide and a difference by chance cannot be ruled out, we think that it may be explained by a so-called healthy traveller effect, which has also been proposed by Kelman and colleagues [5]. This hypothesis proposes that the IR in the absence of travel is lower in a travelling population than in the general population because the former is generally healthier. To assess whether this was the case in our study, we separately calculated the baseline (unexposed) IRs for employees who travelled at least once a year and for those who travelled less than once a year. We found that the baseline IR was indeed lower in employees who travelled more frequently (IRs 0.5/1,000 PY versus 1.2/1,000 PY for those who travelled less). One could therefore argue that only employees who made at least one long-haul flight per year should be used as a reference group, which would have resulted in higher IRRs (which can be inferred from the tables). However, the absolute risks would have remained the same.

Another unexpected finding in this study was the high risk in young travellers. This result may be due to a phenomenon called attrition of susceptibles, meaning that susceptible individuals are likely to develop a disease shortly after start of exposure to a risk factor, such as haemorrhage shortly after start of anticoagulant therapy [18]. Most employees in our cohort had been frequent travellers long before our observation period started. The youngest employees are most likely to be “new frequent travellers,” thus possibly explaining the high absolute risk of thrombosis after air travel in the youngest age category. We assessed whether this was the case as follows: If attrition of susceptibles is present, the baseline IR (i.e., the IR of venous thrombosis without exposure to air travel) in employees who rarely travel (less than once a year) would increase with age. The baseline incidence in employees who fly frequently (more than once a year) would not increase as much with age, since in this group, susceptible individuals would already have suffered from venous thrombosis at a younger age, soon after they became frequent traveller, and hence been excluded from our study population. We found that in our study, the baseline IR in individuals with a low travel-frequency (less than once a year) indeed rose from 0.7/1,000 PY in the youngest age category (<30 y), to 3.2/1,000 PY in the oldest age category (>50 y). In contrast, in individuals with a higher travel frequency, the baseline IR in both the youngest age category and those between 30 and 50 y was 0.8/1,000 PY, whereas no venous thrombosis occurred in 1,521 PY in the oldest age category. Furthermore, in the individuals with a low travel frequency (less than once per year), the incidences in all age groups were very high when they did travel (24/1,000 PY for those under 30 y, 7/1,000 PY for those between 30 and 50 y, and 40/1,000 PY for those over 50 y). So, in these individuals who do not travel on a regular basis, the thrombosis risk is high when they occasionally do. These results all suggest that the risk of air-travel related venous thrombosis is highest in susceptible persons soon after they first start travelling by air, i.e., that attrition of susceptibles is present.

A possible limitation of this study is the response of 32%. Employees who had suffered from venous thrombosis may be more likely to complete the questionnaire than employees who had not. These factors would create bias only if employees who suffered from venous thrombosis directly after air travel responded more frequently than those who suffered from thrombosis without air travel. The response varied considerably per organisation between 15% and 80%. To assess the effect of the response, we analyzed organisations with a low response (≤60%) and those with a high response (>60%) separately. The outcomes did not differ substantially between these two groups of employees, indicating that the low response did not bias our findings. Furthermore, we may have missed employees who died or stopped working due to disability resulting from a venous thrombosis. Although these events are unlikely to have occurred often, it may have led to an underestimation of the number of cases.

Another limitation is that these results cannot be generalized to an older, less-healthy population. This study was performed in a working population with a mean age of 40 y. We do not have any data on people older than age 70 y, nor on individuals who are not fit enough to be employed. Considering these limitations, the absolute risk of venous thrombosis in the general population is likely to be higher than the risk we found. Furthermore, the results cannot be generalized to individuals that have a history of venous thrombosis, as we considered only first events.

From this cohort study among employees of international organisations and companies, we conclude that the absolute risk of symptomatic venous thrombosis within 8 wk of a flight of at least 4 h is approximately 1/4,500 flights. Furthermore, we found three dose–response relationships: the risk of venous thrombosis increased with duration of travel and number of flights a person was exposed to and decreased with time after a long-haul flight. The results of our study do not justify the use of potentially dangerous prophylaxis such as anticoagulant therapy for all long-haul air travellers, since this may do more harm than good [17]. However, for some subgroups of people with a highly increased risk, the risk–benefit ratio may favour the use of prophylactic measures. Large randomized trials are required to assess who would benefit most from which prophylactic measure.

## Abbreviations

BMI: body mass index
CI: confidence interval
IR: incidence rate
IRR: incidence rate ratio
PY: person-year(s)
